# A case of urgent colonoscopic hemostasis of a cecal hemorrhagic ulceration in a patient receiving heparin for COVID‐19 coagulopathy

**DOI:** 10.1002/jgh3.12435

**Published:** 2020-10-23

**Authors:** Naohisa Yoshida, Ryohei Hirose, Makoto Watanabe, Masaski Yamazaki, Satoru Hashimoto, Shin Matsubara, Yu Kasamatsu, Naohisa Fujita, Rafiz Abdul Rani, Osamu Dohi, Ken Inoue, Yuji Naito, Yoshito Itoh

**Affiliations:** ^1^ Department of Molecular Gastroenterology and Hepatology Kyoto Prefectural University of Medicine, Graduate School of Medical Science Kyoto Japan; ^2^ Department of Emergency Medicine Kyoto Prefectural University of Medicine Kyoto Japan; ^3^ Department of Anesthesiology, Division of Intensive Care Kyoto Prefectural University of Medicine Kyoto Japan; ^4^ Department of Infection Control and Laboratory Medicine Kyoto Prefectural University of Medicine Kyoto Japan; ^5^ Gastroenterology Unit, Faculty of Medicine Universiti Teknologi MARA Selangor Malaysia

**Keywords:** colonoscopy, COVID‐19, cytomegalovirus, hemorrhage, personal protective equipment

## Abstract

COVID‐19 rarely causes lower gastrointestinal bleeding even though its RNA has been detected in patient's stool. Urgent colonoscopy in a COVID‐19 patient with massive bloody stool requires various procedural and equipment considerations. Here, we present a case of colonoscopic hemostasis of a cecal hemorrhagic ulceration in a patient on heparin for COVID‐19 coagulopathy. We also share various management methods for the prevention of COVID‐19 contamination. A 71‐year‐old man was diagnosed with COVID‐19 pneumonia and subsequently underwent hemodiafiltration. Heparin was initiated for COVID‐19 coagulopathy. At day 42, the patient experienced 2000 mL of bloody stool. An operator performed urgent colonoscopy with three assistants in a negative‐pressure room with full personal protective equipment. A hemorrhagic ulceration was detected at the cecum, and endoscopic hemostasis was performed. Immunohistochemistry was positive for cytomegalovirus. Postprocedure, the endoscopic systems were thoroughly cleaned, and specific measures for endoscope reprocessing and disinfection were performed to prevent contamination with COVID‐19.

## Graphical abstract

Urgent colonoscopy for a patient with COVID‐19 having massive bloody stool needs various considerations for equipment and procedures. Here, we present the first case report of colonoscopic hemostasis ofcecal hemorrhagic ulceration for a patient receiving heparin for COVID‐19 coagulopathy and also showed various management methods toprevent contamination of COVID‐19.

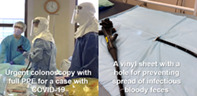

## Introduction

A novel coronavirus, severe acute respiratory syndrome coronavirus‐2 (SARS‐CoV‐2), was first identified in Wuhan, China, and subsequently named COVID‐19 by the World Health Organization.^1^, ^2^, ^3^ The common symptoms of COVID‐19 at the onset of illness are fever, cough, fatigue, myalgia, and dyspnea. SARS‐CoV‐2 enters cells via the angiotensin converting enzyme 2 (ACE2) receptor. ACE2 was highly expressed not only in type II alveolar cells of the lung but also in esophageal epithelial cells and the absorptive enterocytes of ileum and colon, suggesting gastrointestinal (GI) symptoms and possible fecal transmission.^1^, ^3^ A review of the GI symptoms caused by COVID‐19 showed that the incidence ranged from 3% (1/41) to 79% (159/201).^4^ The rate of each symptom reported was as follows: anorexia 39.9% (55/138) to 50.2% (101/201), diarrhea 2% (2/99) to 49.5% (146/295), vomiting 3.6% (5/138) to 66.7% (4/6), nausea 1% (1/99) to 29.4% (59/201), abdominal pain 2.2% (3/138) to 6.0% (12/201), and GI bleeding 4% (2/52) to 13.7% (10/73). GI bleeding may require urgent endoscopy intervention. In addition, recent reports showed many cases of COVID‐19 coagulopathy, which are managed with heparin.^5^ This, in turn, will likely increase the rate of GI bleeding.

SARS‐CoV‐2 is highly contagious as the virus can remain viable and infectious in aerosols for 3 h and on plastic and stainless steel surfaces for up to 3 days.^2^ In addition, given that SARS‐CoV‐2 RNA has been detected in patient's stool, it is possible that SARS‐CoV‐2 could also be transmitted via the fecal–oral route.^6^ Interestingly, even among patients without GI symptoms, SARS‐CoV‐2 RNA was detected in feces (39.1%, 9/23) compared to patients with GI symptoms (52.4%, 22/42). Thus, in reference to these facts, various considerations are required to prevent COVID‐19 infections spreading to medical staff and virus contamination of endoscopic equipment when performing a colonoscopy. Several papers demonstrated the management methods used in endoscopy centers to prevent the spread of the virus.^7^, ^8^, ^9^ However, these guidelines are sometimes inadequate as each country or institution may encounter a new unaddressed situation. To our knowledge, there has been no previous case report on urgent colonoscopy for a COVID‐19 case with massive hemorrhage. Here, we present an urgent case of colonoscopic hemostasis of a cecal hemorrhagic ulceration in a patient on heparin for COVID‐19 coagulopathy, as well as the various management methods for preventing contamination by COVID‐19.

## Case report

**Figure 1:**
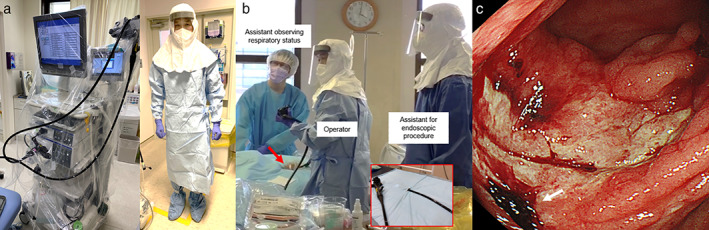
Endoscopic system and personal protective equipment (PPE) for a case of COVID‐19 cecal hemorrhagic ulcer. (a) The endoscopic system was covered by a vinyl chloride sheet prior to being moved to a negative‐pressure room. PPE used when the patient is positive for COVID‐19 or at high risk of it; covering all of exposed skin utilizing an N95 mask, two pairs of gloves, shoe covers, disposable hairnet, disposable face shield, and waterproof disposable gowns. (b) A special blue vinyl chloride sheet with a hole for colonoscope (red arrow and red box) was used to prevent the spread of hemorrhagic feces. The procedure was performed in a negative‐pressure room with an operator, one assistant for the endoscopic procedure, and two assistants observing the respiratory system and patient's status. (c) Cecal ulceration on urgent colonoscopy in a patient with COVID‐19. The exposed vessel was detected under a clot (white arrow) on the ulcer.

A 71‐year‐old man complained of fever, with computed tomography showing bilateral pneumonia. The patient had underlying autoimmune pancreatitis and diabetes mellitus and was on steroids (oral prednisolone: 15 mg/day). The nasopharyngeal swab sample confirmed the presence of SARS‐CoV‐2. The patient was admitted to our hospital and subsequently required artificial respiration. Intravenous prednisolone was prescribed (20 mg/day) instead of oral prednisolone. The patient's condition did not improve even on treatment with favipiravir initially and then on hydroxychloroquine. Hemodiafiltration was started at day 18, and heparin was prescribed for both hemodialysis and coagulopathy secondary to coronavirus. At day 42, the patient suddenly complained of a large volume of bloody stool (approximately 2000 mL). Despite receiving 1400 mL of red blood cell transfusion, the bloody stool continued, resulting in hypovolemic shock. Computed tomography showed diffuse wall thickening of the ascending colon and cecum. Stool cultures were negative, including for *Clostridium difficile*. Heparin was stopped, and we performed an urgent colonoscopy in a specific negative‐pressure room with full personal protect equipment (PPE) to prevent cross‐infection (Fig. 1a,b). The endoscopic system was covered with a sheet of vinyl chloride to prevent contamination. The patient's hip was also covered with a special vinyl chloride sheet to prevent the spread of hemorrhagic feces. The colonoscopy showed ulceration at the cecum and ascending colon (Fig. 1c). An exposed vessel under the clot was detected at the cecal ulceration and treated with endoscopic clips for hemostasis. In addition, heparin was changed to nafamostat mesilate with subsequent cessation of the bloody stool. A blood test and immunohistochemical examination of the biopsy specimen were positive for cytomegalovirus (CMV). The patient was then started on ganciclovir. However, respiratory function and general condition worsened, and unfortunately, the patient died 2 weeks after endoscopic hemostasis. This case report was approved by our institutional ethical board (ERB‐C‐1600).

## Discussion

There are three main issues regarding an urgent colonoscopy for a patient with COVID‐19, as follows: 1. PPE for medical staff due to infectious risk^2^; case‐by‐case management for endoscopic procedures; and.^3^ reprocessing and cleaning of endoscopic systems, various equipment, and rooms.

In treating COVID‐19‐positive patients, PPE is recommended as follows: two pairs of gloves, hairnet, protective face shield, long‐sleeved waterproof gowns, shoe covers, and high‐filter respiratory masks (N95, FFP2, or FFP3).^7^, ^8^, ^9^ We utilized all of the above, with a special hairnet covering the operator's neck. An assistant should help the operator to put on the PPE, and pretraining should be implemented using e‐learning.^7^, ^8^, ^9^ A marketed endoscopic lens cleaner (Cleash, Fujifilm, Tokyo) was applied on the face shield to prevent fogging. The minimum number of endoscopists and assistants should be in the red zone to prevent further infection. We paid attention to possibilities of periendoscopic infections from aerosol particles as the patient had a tracheostomy. Further dispersion might occur by colonoscopic insufflation. Therefore, we wore N95 mask and utilized a special waterproof (vinyl chloride) sheet covering the patient's hip, with a small hole for insertion of the endoscope.

Statements from European Society for Gastrointestinal Endoscopy (ESGE) and European Society of Gastroenterology and Endoscopy Nurses and Associates (ESGENA) showed four categories for endoscopic indications, such as (i) perform always, (ii and iii) case‐by‐case management with high or low priority, and (iv) postpone always.^7^ However, for patients with the coronavirus under artificial respiration, acute lower GI bleeding with hemodynamic instability is the only indication for urgent endoscopy, which is categorized as “perform always” as in our case. In a lower gastrointestinal hemorrhagic case, a paper on COVID‐19 and inflammatory bowel disease (IBD) showed that 3% of patients had CMV colitis in a total of 76 IBD patients.^10^ In our case, steroids prescribed for the patient's autoimmune pancreatitis weakened his immune system, similar to IBD patients. Endoscopists should always consider CMV when a patient on steroid develops lower GI hemorrhage.

In our case, we used single‐use endoscopic accessories, and all were disposed as recommended by several papers.^7^, ^8^, ^9^ Postprocedure, the endoscopic system was wiped in the red zone and moved to the gray zone, and the vinyl chloride sheet was removed. Then, it was moved back to our endoscopic room and wiped clean again. Regarding the endoscope, it was placed in a special box to prevent infections in the gray zone and brought to the washing area of our endoscopic room. It was thoroughly washed by an assistant, who wore gloves, disposable face shield, waterproof disposable gown, and an N95 mask. During flushing of the channel before putting the endoscope in an endoscopic machine wash, we used an N95 mask as, theoretically, aerosolization could occur. Afterward, a standard reprocessing procedure similar to regular disinfection of endoscopy was performed.^7^, ^8^, ^9^ Due to flushing, the floor can be contaminated by droplets and particles. Thus, we disinfected the floor with 1:50 dilution of a household chlorine‐based bleach (final concentration 0.1%, 1000 ppm). The room where the endoscopy was performed was also disinfected with this solution.

Here, we present a case of an urgent colonoscopic hemostasis for a patient with COVID‐19, our tips, and the various management methods used to prevent contamination by COVID‐19.
